# Application Potential of Plant-Derived Medicines in Prevention and Treatment of Platinum-Induced Peripheral Neurotoxicity

**DOI:** 10.3389/fphar.2021.792331

**Published:** 2022-01-13

**Authors:** Xiaowei Xu, Liqun Jia, Xiaoran Ma, Huayao Li, Changgang Sun

**Affiliations:** ^1^ College of First Clinical Medicine, Shandong University of Traditional Chinese Medicine, Jinan, China; ^2^ Oncology Department of Integrative Medicine, China-Japan Friendship Hospital, Beijing, China; ^3^ College of Traditional Chinese Medicine, Shandong University of Traditional Chinese Medicine, Jinan, China; ^4^ Department of Oncology, Weifang Traditional Chinese Hospital, Weifang, China; ^5^ Qingdao Academy of Chinese Medical Sciences, Shandong University of Traditional Chinese Medicine, Qingdao, China; ^6^ College of Traditional Chinese Medicine, Weifang Medical University, Weifang, China

**Keywords:** platinum agents, medicinal plant, phytotherapy, peripheral neurotoxicity, mechanism

## Abstract

As observed with other chemotherapeutic agents, the clinical application of platinum agents is a double-edged sword. Platinum-induced peripheral neuropathy (PIPN) is a common adverse event that negatively affects clinical outcomes and patients’ quality of life. Considering the unavailability of effective established agents for preventing or treating PIPN and the increasing population of cancer survivors, the identification and development of novel, effective interventions are the need of the hour. Plant-derived medicines, recognized as ideal agents, can not only help improve PIPN without affecting chemotherapy efficacy, but may also produce synergy. In this review, we present a brief summary of the mechanisms of platinum agents and PIPN and then focus on exploring the preventive or curative effects and underlying mechanisms of plant-derived medicines, which have been evaluated under platinum-induced neurotoxicity conditions. We identified 11 plant extracts as well as 17 plant secondary metabolites, and four polyherbal preparations. Their effects against PIPN are focused on oxidative stress and mitochondrial dysfunction, glial activation and inflammation response, and ion channel dysfunction. Also, ten clinical trials have assessed the effect of herbal products in patients with PIPN. The understanding of the molecular mechanism is still limited, the quality of clinical trials need to be further improved, and in terms of their efficacy, safety, and cost effectiveness studies have not provided sufficient evidence to establish a standard practice. But plant-derived medicines have been found to be invaluable sources for the development of natural agents with beneficial effects in the prevention and treatment of PIPN.

## 1 Introduction

For platinum-based chemotherapy agents were used for cancer treatment in the 1970s, and have been considered one of the most prescribed and effective anticancer agents ever developed (Rosenberg and VanCamp, 1970). However, only cisplatin (first-generation product), carboplatin (second-generation product), and oxaliplatin (third-generation product) are used worldwide for cancer treatment, and certain platinum-based drugs (nedaplatin, lobaplatin and heptaplatin) are only approved in individual countries or regions (Dilruba and Kalayda, 2016; Oun et al., 2018). Currently, these drugs continue to be extensively used in the treatment of cancer and play an important role in changing the natural history of many cancer types (Kelland, 2007). Unfortunately, as observed with other anticancer agents, platinum-based compounds exert several acute and chronic toxic side effects that often limit their clinical use. The induction of severe PIPN is a grave dose-limiting side effect. PIPN includes acute and chronic peripheral neurotoxicity, with the latter being observed and associated with the administration of platinum-based compounds, without restriction to any agent, while the former being restricted to and observed in cases of oxaliplatin administration. Regarding PIPN, the peripheral neurotoxicity of carboplatin is negligibly compared to that observed with oxaliplatin and cisplatin administration (Stankovic et al., 2020). Recent long-term clinical and nerve conduction studies (NCS) have suggested that chronic PIPN with complete reversion occurs only in a few cases (Kokotis et al., 2016). Additionally, a phenomenon known as the “the coasting effect,” which demonstrates signs and symptoms of PIPN, can appear or even worsen after treatment cessation (Briani et al., 2014). PINP, recognized as one of the most important complications of subjection to contemporary tumor therapeutic strategies, not only limits the further use of curative-intent treatment, but also leads to the development of issues pertaining to the long-term quality of life of the afflicted individuals. Currently, the prolonged survival time of cancer patients is attributed to advances in diagnosis and treatment, thus garnering attention in terms of the management of health-related quality of life in follow-up care. Therefore, PIPN has gained prominence as a relatively common emergency issue warranting investigation to confer protection and to sustain the quality of life of patients. Moreover, PIPN poses significant burden on the annual health care budget, and such a burden may be long-lasting or permanent (Pike et al., 2012).

Although PIPN has been known for several decades, the management of PIPN continues to be challenging. During this period, adjuvants for the prevention or treatment of PIPN have been widely developed in preclinical studies. However, according to the most recent guidelines reported in the year 2020 by the American Society of Clinical Oncology (ASCO) Prevention and Management of Chemotherapy-Induced Peripheral Neuropathy (CIPN) in Survivors of Adult Cancers, no agents can be recommended for the prevention of CIPN due to lack of high-quality, relevant evidence (Loprinzi et al., 2020), an aspect which is the same as reported in the initial 2014 guidelines (Hershman et al., 2014). Thus far, only duloxetine has been reported as an effective therapeutic agent for treating oxaliplatin-induced painful peripheral neuropathy (Smith et al., 2017). Hence, we aimed to establish novel interventions to prevent the occurrence of peripheral nerve injuries during earlier phases, rather than establishing after the development of neurological deficits. Therefore, it is necessary to formulate more effective prevention and treatment strategies in the presence for clinical application constraint of PIPN adjuvants.

A significant number of plant-derived medicines have been generally recognized as potential drug candidates for the prevention and treatment of PIPN, and a continuous increase in the number of such candidates has been documented with successive research (Newman and Cragg, 2020). Plant-derived medicines, as a paradigm of proactive medicine, exert fewer side effects, and a few patients have used or expressed interest in their usage to prevent diseases and/or to improve the quality of life; additionally, such medicines have attracted the attention of scientists (Santini et al., 2017). The effectiveness of phytocomplexes for the management of PIPN has also been supported by new scientific evidence reported in preclinical and clinical studies (Lee and Kim, 2016; Ebrahimi et al., 2019). In this context, the search for phytocomplexes may help provide a steady and valuable source of adjuvants to improve PIPN.

In this review, the potential intervening mechanisms of PIPN adopted by the plant extracts, plant secondary metabolites, and polyherbal preparations have been discussed and include oxidative stress and mitochondrial dysfunction, glialactivation and inflammatory response, and ion channel dysfunction. The complex nature of the underlying mechanisms evidently exclude the possibility of using a single molecule to eliminate PIPN, since most natural products present with multiple targets and may involve the participation of more than one signaling pathway (Cheng et al., 2015). This provides insights for researchers aiming to ensure that the anticancer activity of platinum agents is not weakened viaco-administration with natural products, and that the approachis beneficial in reducing patient peripheral neurotoxicity. Owing to their multitarget, multilevel, convenient, economical, effective, and relative safe integrated benefits, the use of natural products seems to be a feasible strategy for the management of PIPN (Schröder et al., 2013; Lee and Kim, 2016).

## 2 Search Strategy

Electronic databases including PubMed and Web of Science were searched using the following keywords:(“chemotherapy-induced peripheral neuropathy” OR “Cisplatin” OR“Oxaliplatin” OR “Carboplatin” OR “Platinum compounds”) AND (“peripheral neuropathy” OR “CIPN”OR “PIPN”) AND (“herb” OR “plant” OR “extract” OR “herbal medicines” OR “Chinese herbal medicines” OR “ayurvedic herbal medicines”). Data were collected from inception until August 2021. Only papers with English full-text were included in our study. Studies on the neuropathies due to causes other than platinum compounds chemotherapy were excluded. Studies on the other side effects of platinum compounds chemotherapy were also excluded. From total of 2094 studies, final number of 46 papers, including 36 animal studies and 10 clinical trials, were retrieved and summarized systematically. Results of the final included article are summarized in Table 1 and Table 2.

**TABLE 1:** Compilation of experimental data related to protective effect of plant-derived medicines against PIPN.

Plant-derived medicines	Pt-based drug	Study model	Dose, route, and duration of administration	The dose range	The minimal active concentration	Type of extract used	Reported method to obtain the extract/use of a standardized extract	Controls	Mechanism and effect	Ref
Extract of *Forsythia viridissima* fruits	Oxaliplatin	Male mice (C57BL/6) and male Sprague-Dawley rats	Administration of 100 mg/kg, 6 times per week, for 5 weeks	50–100 mg/kg	50 mg/kg	Aqueous extraction	Yes	Vehicle control	Rescues DRG cells from the oxaliplatin-induced mitochondrial membrane depolarization and reverses the oxaliplatin-induced apoptosis	Yi et al. (2019a)
Extract of *Forsythia suspensa* fruits	Oxaliplatin	Neural PC12cells	Administration of 0–100 μg/ml for 48 h; administration of 250 mg/kg/day for 3 weeks	0–400 μg/ml	100 μg/ml	Aqueous extract	Yes	Vehicle control	Affects the oxaliplatin-induced neuroinflammation, oxidative stress, mitochondrial dysfunction, or axonal degeneration	Yi et al. (2019b)
		Male mice (C57BL/6)		60–600 mg/k g	60 mg/kg					
*Ginkgo biloba* extract -EGb761	Cisplatin	Female Swiss albino mice	Administration of 100 mg/kg, twice a week, for a total of nine injections over 4.5 weeks	100 mg/kg	100 mg/kg	Standardized extract	Yes	Vehicle control	Provides antioxidant protection for primary sensory neurons, prevents the decrease of NCV caused by cisplatin and the decrease of exogenous axon length	Oztürk et al. (2004)
*Vitis vinifera* extract	Oxaliplatin	Male sprague-dawley rats	Administration of 300 mg/kg, for 5 consecutive days, every week for 3 weeks	300 mg/kg	300 mg/kg	Hydroalcoholic extract	Yes	Vehicle control	Reduces oxaliplatin-dependent superoxide anion increase and lipid peroxidation in rat astrocytes	Micheli et al. (2018)
*Hypericum perforatum* extract	Oxaliplatin	Astrocyte and HT-29 cells	Administration of 5, 50, 250 μg/ml for 4 and 8 h	5–250 μg/ml	5 μg/ml	Hydrophilic extract	Yes	Vehicle control	Reduces caspase-3 activity in rat astrocytes; antioxidant effect	Cinci et al. (2017)
*Astragalus mongholicus* extract	Oxaliplatin	Male sprague-dawley rats	Aqueous (Aqu) and 20% hydroalcoholic (HA) administered at a dose of 300 mg/kg/day, for 3 weeks; 50%HA administered in a dose range of 30–300 mg/kg/day, for 3 weeks	300 mg/kg	300 mg/kg	Aqueous and hydroalcoholic extract	Yes	Vehicle control	Reduces the enhancement of caspase-3 activity, decreases the oxaliplatin-dependent oxidative stress of lipids, proteins, and nucleic acid	Di Cesare Mannelli et al. (2015); Di Cesare Mannelli et al. (2017b)
*Agrimonia eupatoria* extract	Cisplatin	Male sprague-dawley rats	Administration of 200 mg/kg for 1 week	200 mg/kg	200 mg/kg	80% ethanol extract	Yes	Positive control (gabapetin)	Demonstrates an antinociceptive effect in the pin-prick test, plantar test, and paw-withdrawal threshold test using a cisplatin-induced neuropathic rat model	Lee and Rhee, (2016)
Extract of *Lithospermum erythrorhizon*	Oxaliplatin	PC12cells	Administration regimen:Low, 25 μg/ml; High, 100 μg/ml; for 3 days	25–100 μM	25 μM	Aqueous extract	Yes	Vehicle control	Exerts an anti-inflammatory activity in neuronal immune cells	Cho et al. (2016)
		male C57BL/6 mice	Administration of 250 mg/kg, 6 days per week	250 mg/kg	250 mg/kg					
*Salvia officinalis* extract	Cisplatin	NMRI male mice	Administration of 100 mg/kg, i.p.every 24 h, for 96 h	100 mg/kg	100 mg/kg	Aqueous-alcoholic extract	YES	Positive control (morphine)	Inhibits the molecular targets of pro-inflammatory mediators PGE2 in inflammatory responses	Namvaran-Abbas-Abad and Tavakkoli, (2012)
*Matricaria chamomilla* Hydroalcoholic extract	Cisplatin	NMRI male mice	Administration of 25 mg/kg every 24 h, for 96 h, IP	25 mg/kg	25 mg/kg	Hydroalcoholic Extract	Yes	Positive control (morphine)	Decreases production of cytokines from lipopolysaccharides *in vivo* and *in vitro* and controls inflammation	Abad et al. (2011)
*Aconitum carmichaelii* extract	Oxaliplatin	Male sprague-dawley rats	Administration of 300 mg/kg/day, for 5 days	300 mg/kg	300 mg/kg	Diluted with distilled water	Not	Vehicle control	Suppresses the activated spinal astrocytes and downregulates expression of proinflammatory cytokines (IL-1β and TNF-α)	Jung et al. (2017)
*Camellia sinensis* extracts	Oxaliplatin	Male sprague-dawley rats	Administration of 300 mg/kg/daily for 6 weeks	300 mg/kg	300 mg/kg	Commercially available compounds	Not	Vehicle control	Alleviates oxaliplatin administration produces an acute functional channelopathy of axonal Na + channels	Lee et al. (2012)
Curcumin	Cisplatin	Female wistar rats	Administration of curcumin 200 mg/kg/day for 5 weeks	200 mg/kg	200 mg/kg	Commercially available compounds	NA	Vehicle control	Reduces oxidative stress caused by elevated ROS level and mitochondrial dysfunction	Agthong et al. (2015)
Curcumin	Oxaliplatin and cisplatin	Male wistar rats	Administration of 10 mg/kg, twice, weekly, for 4.5 weeks	10 mg/kg	10 mg/kg	NA	NA	Vehicle control	Reverses the alterations in the plasma neurotensin and sciatic nerve platinum concentrations, and improves sciatic nerve histology in the platinum-treated rats	Al Moundhri et al. (2013)
Rutin and quercetin	Oxaliplatin	Male Swiss mice	Administration of rutin (25, 50, and 100 mg/kg), quercetin (25, 50, and 100 mg/kg), twice a week (on Mondays and Thursdays) with a total of nine doses administered	25–100 mg/kg	25 mg/kg	NA	NA	Vehicle control	Inhibits oxaliplatin induced oxidative stress and nitric oxide and peroxynitrite the effect in the spinal cord	Azevedo et al. (2013)
Formononetin (FN)	Oxaliplatin	Mouse ND7/23 neuroncells	0.1,1,10,25 μM) for 0–48 h; administration of 10 mg/kg, dissolved in corn oil, withintraperitoneal injection for 5 days, followed by 5 days of rest, for two weekly cycles	0.1–25 μM	0.1 μM	NA	NA	Vehicle control	Promotes cell survival and prevents mitochondrial dysfunction and apoptosis through the activation of the NRF2 pathway and its downstream-GSTP1	Fang et al. (2020)
		C57BL/6 male mice		10 mg/kg	10 mg/kg					
Cyanidin	Cisplatin	PC12 cells	Administration of 10–80 μM cyanidin for 24 h	10–80 μM	10 μM	Commercially available compounds	NA	Vehicle control	Inhibits DNA damage, attenuates p53 phosphorylation, and eventually reverses cell apoptosis through inhibition of ROS accumulation	Li et al. (2015)
Silibinin	Oxaliplatin	Male sprague-dawley rats	Administration of 100 mg/kgper os, once a day, for 20 days	100 mg/kg	100 mg/kg	Commercially available compounds	NA	Vehicle control	Protects astrocyte from the oxaliplatin induced extrinsic apoptosis; reduces the oxidative stress, free radical scavenging	Di Cesare Mannelli et al. (2012)
Rosmarinic Acid (RA)	Oxaliplatin	N2acells	Administration of RA 50 μM for 24 h	50 μM	50 μM	NA	NA	Vehicle control	Reduces the oxidative stress, improves the mitochondrial function, activates AMPK in peripheral nerves and DRG	Areti et al. (2018)
		Male sprague-dawley rats	Administration of RA 25 and 50 mg/kg for 28 days	25–50 mg/kg	25 mg/kg					
Astragaloside IV(AS-IV)	Oxaliplatin	Male sprague-dawley rats	Low, medium, and high AS-IV groups subjected to a daily gavage of AS-IV 10, 20, or 40 mg/kg body weight for 4 weeks, respectively	10–40 mg/kg	10 mg/kg	Commercially available compounds	NA	Vehicle control	Reduces TNF-α, IL-6, and IL-1β to inhibit inflammation; decreases MDA, raised SOD, CAT, and GSH- Px in the spinal cord to block oxidative stress	Xu et al. (2021)
TanshinoneIIA	Oxaliplatin	N2a cells	Administration of TanshinoneIIA 0–20 μM for 24 h	0–20 μM	1 μM	Commercially available compounds	NA	Vehicle control	Prevents excessive oxidative stress via reduction of ROS levels and Ψm loss; protects mitochondria via reduction of mitochondrial membrane potential loss; promotes autophagy through the PI3K/Akt/mTOR signaling pathway	Cheng et al. (2019)
		Male sprague-dawley rats	Injection with tanshinoneIIA 25 mg/kg/day, for 7 days	25 mg/kg	25 mg/kg					
Thymoquinone (TQ)	Cisplatin	BALB/c mice	Administration of 0.025, 0.05, 0.1, 1, 10, 25, 50 μM for 72 h	0.025–50 μM	0.025 μM	Commercially available compounds	NA	Vehicle control	Reduces oxidative stress status via the potent anti-oxidant and free radical scavenging action; inhibits the apoptotic cascade (increasing Bcl-2 expression, repressing the activation of caspase-9 and caspase-3 and reducing the cleavage of PARP- 1	Üstün et al. (2018)
Ergothioneine	Oxaliplatin	Male sprague-dawley rats	Administration of 1.5 mg/kg,twice/week, for 6 weeks	0–15 mg/kg	1.5 mg/kg	Commercially available compounds	NA	Vehicle control	Decreases the accumulation of OCTN1 and oxidative stress in DRG neurons	Nishida et al. (2018)
Alpha-lipoic acid	Cisplatin	Rats (not specified)	Administration (concentration range: 1 μM–1 mM) for 3 h	1–1,000 μM	1 μM	—	NA	Vehicle control	Induces the expression of frataxin, prevents axonal damage, apoptosis, and mitochondrial energetic failure in sensory neurons	Melli et al. (2008)
6-Methoxyflavone	Cisplatin	BALB/c mice and male sprague-dawley rats	Daily treatment with 6-MF (25, 50, and 75 mg/kg/day, i.p.) for 4 weeks	25–75 mg/kg	25/kg	Commercially available compounds	NA	Vehicle control	Mediates through inhibition or activation of local peritoneal receptors or inhibition of COX-1 and COX-2	Shahid et al. (2017)
Ginsenoside F2	Oxaliplatin	PC12 cells	Administration of 6.25 ,12.5, and 25 μM for 24 h	6.25–25 μM	6.25 μM	Commercially available compounds	NA	Vehicle control	Prevents oxaliplatin-induced reduction in neurite-like growth in differentiated PC12 cells	Suzuki et al. (2015)
Coumarin	Oxaliplatin	Male sprague-dawley rats	Administration of 10 mg/kg for 5 days	10 mg/kg	10 mg/kg	Aqueous extract	NA	Vehicle control	Suppresses the activation of astrocytes and microglia and decreases the expression levels of IL-1β and TNF	Kim et al. (2016)
Cinnamic acid	Oxaliplatin	Male sprague-dawley rats	Administration of 10, 20, and 40 mg/kg (i.p.) in rats on day 4	10–40 mg/kg	10 mg/kg	Commercially available compounds	NA	Vehicle control	Inhibits glial and/or cytokines (IL-1β and TNF) activation	Chae et al. (2019)
Ginsenoside Rg3	Oxaliplatin	Male ddY mice	Administration of ginseng extract 0.2 g/kg/day, from days 0–6	10 μM	10 μM	Commercially available compounds	NA	Vehicle control	Stabilizes excitable cells by preventing the influx of cations such as Ca (2+) and Na (+)	Suzuki et al. (2017)
Neoline	Oxaliplatin	Male ddY mice	Administration of 0, 0.25, 0.5, 1,2 μg/ml for 48 h	0–2 μM	0.25 μM	Aqueous extract	NA	Vehicle control	Inhibits the nociceptive transmission and spinal glial activation following peripheral nerve damage	Suzuki et al. (2016)
*Corydalis saxicola* Bunting total alkaloids	Cisplatin	Male sprague-dawley rats	Administration of 30, 60, 120 mg/kg twice a week for 5 weeks	30–120 mg/kg	30 mg/kg	Commercially available compounds	NA	Vehicle control	Ameliorates neuronal damages and IENF loss, and inhibits pro-inflammation cytokines (TNF-α,IL-1β,PGE2)-induced p38 phosphorylation to block the TRPV1 activation	Kuai et al. (2020)
Glucoraphanin (GRA) and sulforaphane (SFN)	Oxaliplatin	Male CD-1 albino mice	Administration of GRA4.43, 13.31, 39.93, and 119.78 μmol kg^−1^ for 14 days	GRA 4.43–119.79 μmol kg^−1^	GRA 4.43 μmol kg^−1^	Purified at the laboratory	NA	Vehicle control	Reduces neuropathic pain by releasing H2S and modulating Kv7 channels	Lucarini et al. (2018)
			Administration of SFN 1.33, 4.43, and 13.3 μmolkg^−1^ for 14 days	SFN 1.33–13.31 μmolkg^−1^	SFN 1.33 μmolkg^−1^					
AC591 (extract of HGWD)	Oxaliplatin	Male wistar rats	Administration of 5 g, 10 g, 20 g/daily for 4 weeks	5–20 g/kg	5 g/kg	Aqueous extract	Yes	Vehicle control	Depends on modulation of multiple molecular targets and pathways involved in the downregulation of inflammatory cytokines (IL-1β, IL-6 and TNF-α) and immune response	Cheng et al. (2017)
Gyejigachulbu-tang (GBT)	Oxaliplatin	Male sprague-dawley rats	Administration of 200, 400, and 600 mg/kg/day for 5 days	200–600 mg/kg	200 mg/kg	Commercially available compounds	NA	Vehicle control	Restores immune activities of GFAP and OX-42, suppresses the activation of spinal astrocytes and microglia	Ahn et al. (2014)
DangguiSini decoction (DSD)	Oxaliplatin	SPF wistar rats	Administration of 10 mg/kg daily for 4 weeks	6.2–4.8 g/kg	6.2 g/kg	NA	NA	Vehicle control	Reduces the current amplitude of DGR cells undergoing agonists stimuli (TRPV1,TRPM8, and TRPA1 agonist), suppresses inflammatory lesions, improves ultra-microstructures, and increases the number of Nissl bodies	Ding et al. (2020)
Goshajinkigan (GJG)	Oxaliplatin	Male wistar-ST rats	Oral dose of GJG, 1 g/kg/day for 12 days	0.3–1.0 g/kg	0.3 g/kg	Commercially available compounds	NA	Vehicle control	Suppresses the functional alteration of TRP channels, especially TRPA1 and TRPM8	Kato et al. (2014)

*Additional abbreviations:* NA, not apply.

**TABLE 2:** Clinical evidence on the effectiveness of plant-derived medicines in platinum-induced peripheral neurotoxicit.

Plant-derived medicines	Dosage	Study design	Jadad score	Outcome	Ref
Goshajinkigan (GJG)	7.5 g/day	Retrospective study in 90 metastatic colorectal cancer patients with oxaliplatin-induced peripheral neuropathy	—	Concomitant administration of Goshajinkigan reduced the neurotoxicity of oxaliplatin in patients that received chemotherapy for colorectal cancer	Kono et al. (2011)
Goshajinkigan (GJG)	7.5 g/day	Retrospective study in 45 non-res ectable or recurrent colorectal cancer patients with oxaliplatin-induced peripheral neuropathy	—	Goshajinkigan is useful in preventing oxaliplatin-induced neuropathy in patients with non-resectable or recurrent colorectal cancer	Nishioka et al. (2011)
Goshajinkigan (GJG)	7.5 g/day	Retrospective study in 73 colorectal cancer patients with oxaliplatin-induced peripheral neuropathy	—	Goshajinkigan prevented exacerbation of oxaliplatin-induced peripheral neuropathy	(Yoshida et al., 2013) Naohisa Yoshida
Goshajinkigan (GJG)	7.5 g/day	A phase 2, multicenter, randomized,double-blind,placebo-controlled trial in 93 colorectal cancer patients	5	Oral Goshajinkigan has acceptable margins of safety and tolerability and a promising effect in delaying the onset of grade 2 or greater OIPN in colorectal cancer patients treated with oxaliplatin	Kono et al. (2013)
Goshajinkigan (GJG)	7.5 g/day	Placebo-controlled, double-blind, randomized phase III study in 142 colon cancer patients	5	Goshajinkigan did not prevent oxaliplatinassociated peripheral neuropathy in this clinical trial	Oki et al. (2015)
Guilongtongluofang	200 ml/day	Randomized, Double-Blind, Placebo-Controlled Trial in 120 colorectal cancer patients with adjuvant oxaliplatin-based chemotherapy	5	Guilongtongluofang is useful in preventing acute and chronic oxaliplatin-induced neurotoxicity in patients with colorectal cancer	Liu et al. (2013)
ninjin’yoeito	9.0 g/day	Randomized, open-label, phase 2 trial in 52 patients with colorectal cancers of pathological stage 3	3	Ninjin’yoeito has prophylactic efficacy against oxaliplatin-induced cumulative peripheral neuropathy in patients with colorectal cancers	Motoo et al. (2020)
*Lawsonia inermis* L	50 g of pure henna plant powder were applied before going to bed (for 8–10 h on average)	Parallel-group, randomized, controlled pilot trial in 60 female patients receiving oxaliplatin-based treatment	4	*Lawsonia inermis* application on hands and feet has a beneficial effect on peripheral neuropathy	Arslan et al. (2020)
AC591	54 g/day	Randomized, Double-Blind, Placebo-Controlled Trial in in 72 colorectal cancer patients	5	AC591 can prevent oxaliplatin-induced neuropathy without reducing its antitumor activity	Cheng et al. (2017)
Jiawei Huangqi Guizhi Wuwu Decoction (JHGWD)	200 ml/day	Randomized con- trolled self-crossover trial in 31 patients with gastric carcinoma and colorectal carcinoma	2	Jiawei Huangqi Guizhi Wuwu Decoction could prevent acute neurotoxicity and mitigate the adverse reaction induced by oxaliplatin	Li et al. (2006)

## 3 Potential Mechanisms

**FIGURE 1:**
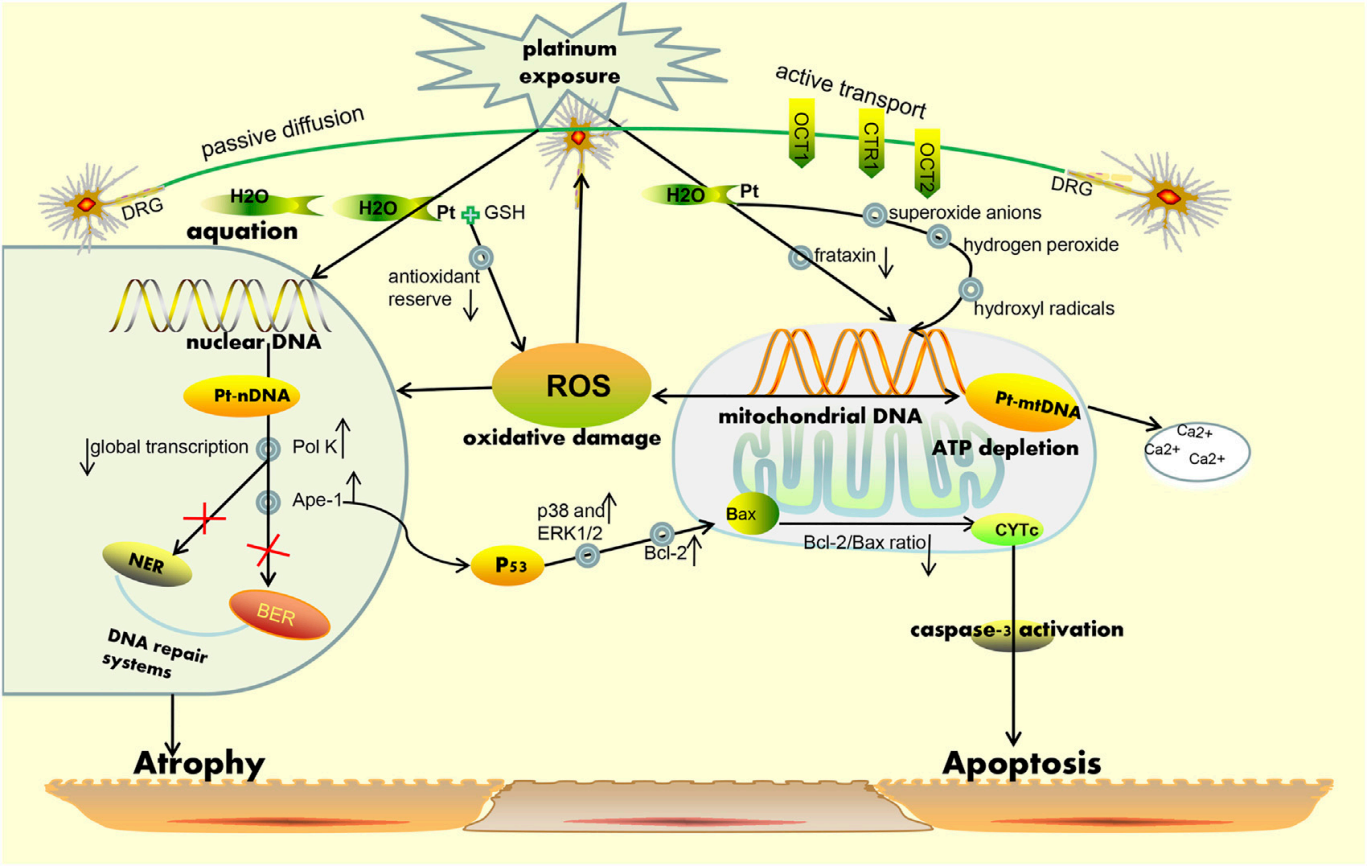
Simplified schematic representation of the mechanism for platinum-induced peripheral neurotoxicity hypothesis. On one hand, platinum-based drugs are subjected to uptake by the sensory neuron cells *via* passive diffusion through the plasma membrane, and on the other hand, active transport is necessary through the copper transporters OCT1, OCT2, and CTR1for entry into the cell. Correspondingly, when platinum-based agents enter the neuron, they become reactive viaaggressive hydration and can also respectively bind to nuclear and mitochondrial DNA regions. Platination of the nuclear DNA may cause the increase in the expression of Ape-1 and pol K protein, leading to the occurrence of an inefficient DNA damage repair system (i.e., BER and NER pathways) and a decrease in general transcription. In parallel, Ape-1 protein also results in the activation of p53, following which activated p53induces the release of cytochrome c (CytC) from the mitochondria and subsequent caspase-3 activation. All above mentioned phenomena may cause neuronal death due to apoptosis. Furthermore, the binding of platinum-based drugs to the mitochondrial DNA may induce the decrease of replication and lead to a failure in overall function at the mitochondrial level. This eventually causes depletion of ATP and an increase in ROS formation as well as sequestration of intracellular calcium. Notably, the mitochondria are considered the main sources of ROS production and are recognized as the major targets for ROS-induced oxidative damage, a phenomenon which can lead to the reduction of the efficiency of mitochondria and induction of apoptosis. *Additional abbreviations:* CTR, copper transporters; OCT, organic cation transporters; NER, nucleotide excision repair; BER, base excision repair; Pol k, polymerase kappa; Ape-1, apyrimidinic endonuclease/redox effector factor.

**FIGURE 2:**
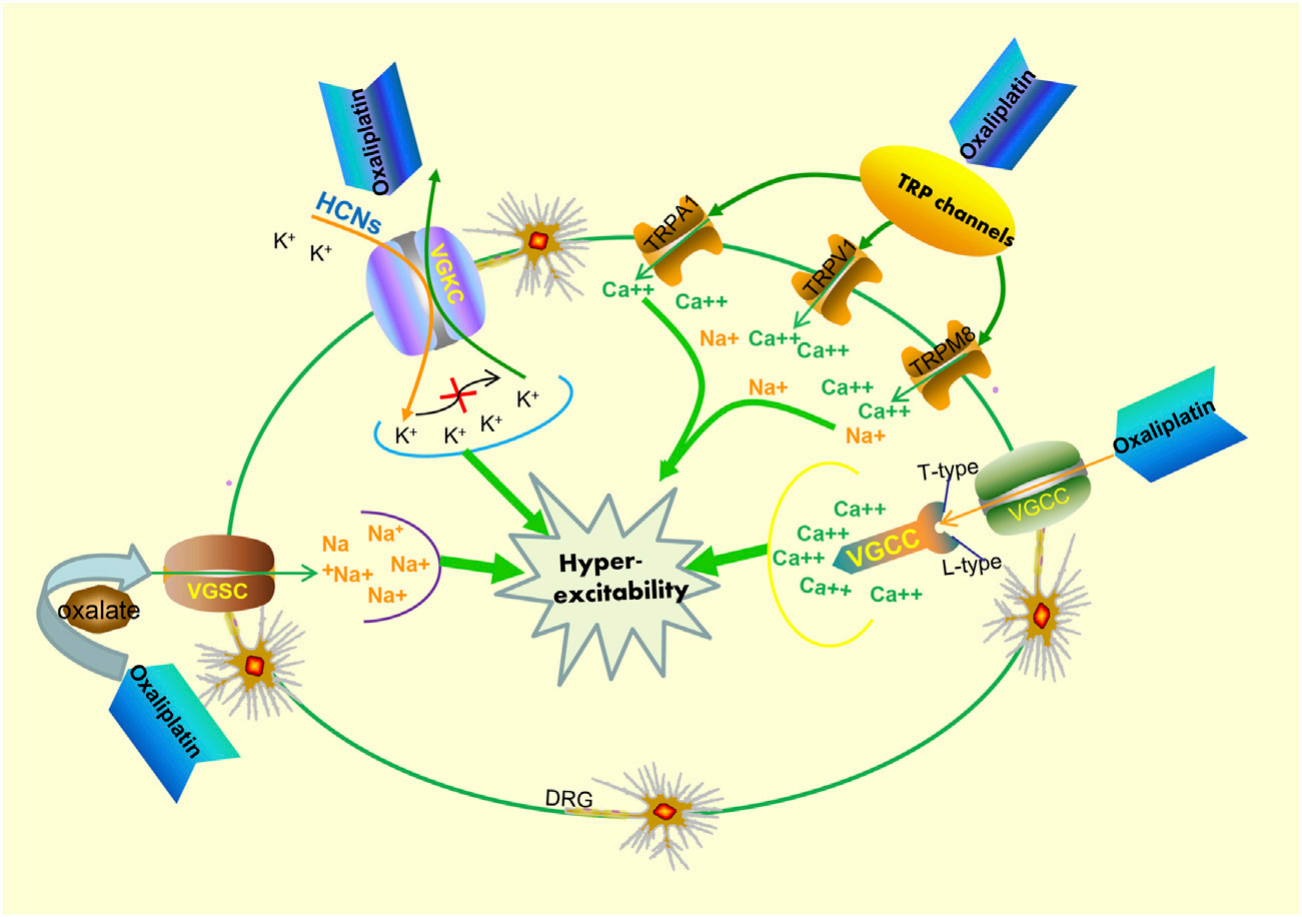
Potential mechanisms of acute oxaliplatin-induced peripheral neurotoxicity. Oxaliplatin exposure can respectively affect the activity and kinetics of both voltage-gated sodium channels (VGSC) and voltage-gated potassium channels (VGKC). On one hand, oxaliplatin exposure can influence the functional properties of VGSC, resulting in hyperexcitability of DRG sensory neurons. On the other hand, oxaliplatin exposure can also lead to functional abnormalities of VGSC and improve cell excitability byincreasing hyperpolarization-activated channel (HCNs) expression. Additionally, the transcription levels of T- and L-type voltage-gated calcium channels (VGCC) increase after oxaliplatin exposure, resulting in the dysregulation of Ca^2+^ homeostasis. Oxaliplatin exposure also leads to an upregulation of the sensitization of the TRPV1, TRPA1, and TRPM8 in cultured DRG neurons, and this occurrence plays a pivotal role in the neuronal hyperexcitability phenomenon.

**FIGURE 3:**
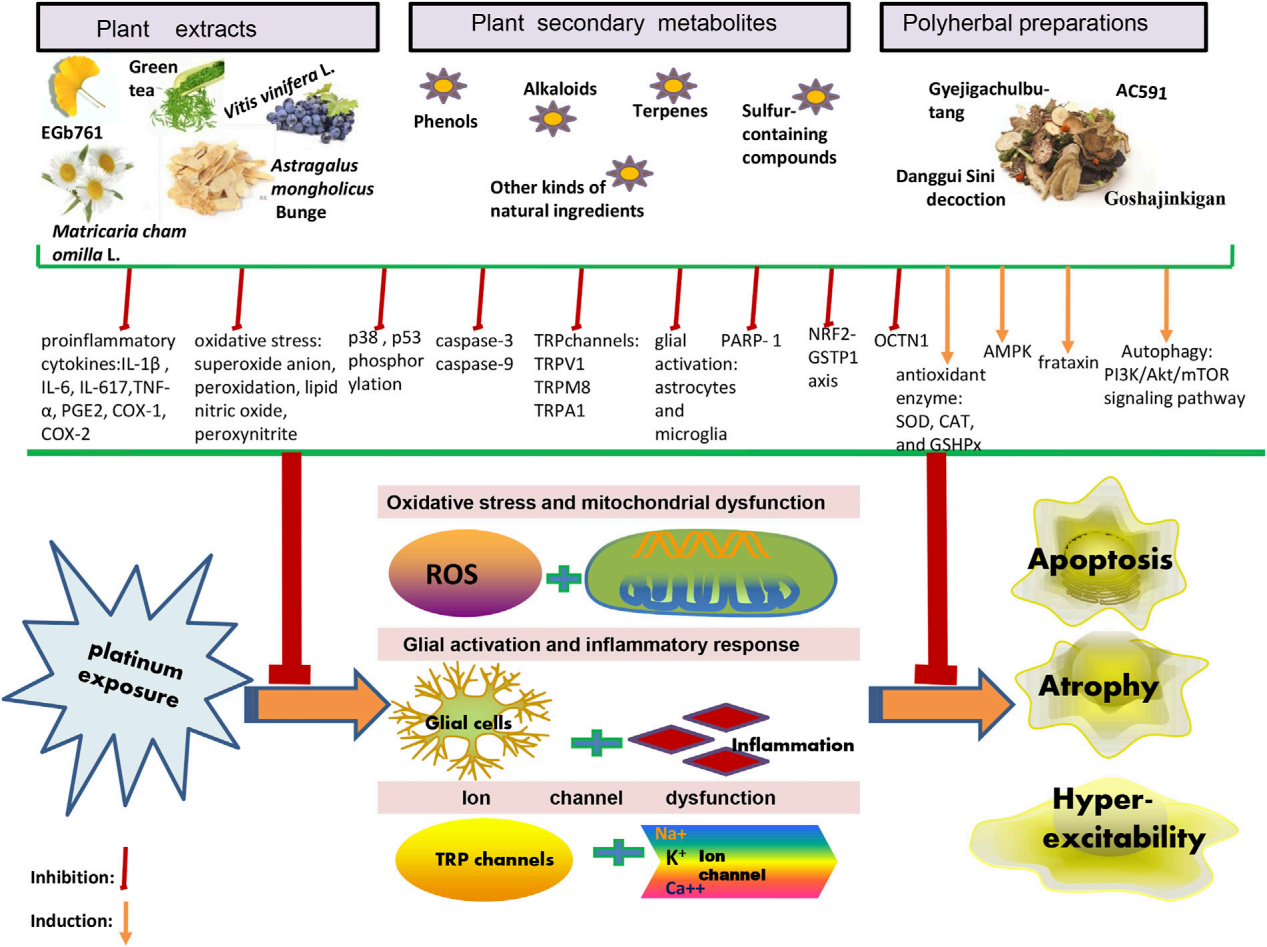
Schematic mechanisms of medicinal plants to prevent PIPN.

Owing to the complex interactions established in the sensor cells, neurons, glia, and effector cells, platinum agents can induce peripheral neuropathyin a different manner (Kang et al., 2021). A concise summary of the cellular and molecular mechanisms of PIPN is presented in Figure 1 and Figure 2. Remarkably, the mechanisms of acute and chronic Oxaliplatin-induced peripheral neuropathy (OIPN) are not the same. Schematic mechanisms of medicinal plants to prevent PIPN is presented in Figure 3.

The mechanisms for PIPN are multifactorial (Staff et al., 2019; Calls et al., 2020), and there is a lack of comprehensive information on the mechanisms involved. The absence of information on the pathogenetic mechanisms also leads to a lack of availability of valid treatments (Alberti, 2019). Earlier studies have shown that the cytotoxic mechanism of action of platinum agents and their action on cells in the peripheral nervous system cells share several important similarities (Kanat et al., 2017; Schloss et al., 2017). The mechanism of action of platinum drugs involves four main steps, namely cellular uptake, hydration and activation, DNA platination, and transcription inhibition (Ang et al., 2010). Based on the remarkable efforts engaged in preclinical studies, many different hypotheses have been suggested to explain PINP pathogenesis. The main common mechanisms of pathogenesis include mitochondrial dysfunction, oxidative stress, DNA damage, and apoptosis induction (McDonald et al., 2005; Alaedini et al., 2008). Notably, mitochondrial DNA is not protected by the physiological mechanisms of DNA repair systems. At the protein level, platinum-based compounds demonstrate a high affinity to mitochondrial DNA, which is no lesser affinity than to nuclear one. For this reason, mitochondrial DNA is more susceptible than nuclear DNA to platinum compound-induced damage (Hetman et al., 2010; Podratz et al., 2011). Researchers continue to recognize and report the importance of mitochondria in mediating neuroprotection. Further studies demonstrated that mitochondrial dysfunction could lead to an increase in reactive oxygen species (ROS)and oxidative stress (Podratz et al., 2017). Mitochondria are also considered a primary target of ROS-induced damage (Chen et al., 2019). Additionally, acute oxaliplatin-induced neurotoxicity is a remarkable phenomenon. Currently, the most accredited hypothesis for adjunctive pathogenetic pathways is the hyperexcitability of sensory neurons (Alberti, 2019).

The potential mechanisms of oxaliplatin-induced acute neurological side effects are essentially based on the following two phenomena: 1) impaired activity of voltage-gated sodium (Na^+^), potassium (K^+^), and calcium (Ca^2+^) ion channels (Kagiava et al., 2008; Chiorazzi et al., 2015; Schmitt et al., 2018),and/or 2) the increased sensitivity of transient receptor potential (TRP) channels in sensory neurons (Marmiroli et al., 2015). Additionally, the results of a randomized phase III trial showed that a possible correlation between the severity of acute oxaliplatin neurotoxicity and susceptibility to chronic neuropathy could exist, albeit through different mechanisms of toxicity (Pachman et al., 2015). However, the binding of platinum drugs to the mitochondrial DNA may induce sequestration of intracellular calcium, and this seems to provide a plausible explanation for the correlation observed between the acute and chronic peripheral neurotoxicity pertaining to oxaliplatin use. Specifically, the transcription levels of T-andL-type voltage-gated calcium channels increase after cells are exposed to oxaliplatin, which subsequently induces an abnormal accumulation of calcium. Collectively, it is evident that a single unifying mechanism does not exist that may be considered to elucidate PIPN development.

In recent years, with a deeper understanding of PIPN, data on complex mechanisms, such as glial activation, inflammatory response, and ion channel dysfunction, are constantly being updated (Di Cesare Mannelli et al., 2013a; Di Cesare Mannelli et al., 2014). The rapid development of molecular biology techniques has helped obtain a new out look on PIPN. The identification of predictive markers for patients with respect to symptoms, responses, and side effects may help in future developments in the field (Dilruba and Kalayda, 2016). The aim of this study was to comprehensively review the efforts engaged in various studies conducted on the use of phyto complexes in alleviating PIPN, while the effectiveness and related mechanisms for the protection of peripheral nerve cells have been highlighted. Based on the existing information, we hope to formulate new therapeutic strategies, to provide insights into the treatment and prevention of PIPN, and to improve the patient’s quality of life, while maintaining the comprehensive anticancer efficiency of platinum-based agents.

## 4 Plant-Derived Medicines Limiting Platinum-Induced Peripheral Neurotoxicity

In this section, we have systematically described the phytocomplexes and medicinal plants investigated for their neuroprotective effects against PIPN. Depending on the different investigated studies, we systematically divided the information into three parts, namely 1) plant extracts, 2) plant secondary metabolites, and 3) polyherbal preparations. Therein, the plant secondary metabolites are further divided into phenols, alkaloids, terpenes, sulfur-containing compounds and other kinds of natural ingredients according to our studies. The structure of the plant secondary metabolites were list in Supplementary Table S1 as supplementary material.

### 4.1 Plant Extracts

#### 4.1.1 *Forsythia suspensa* (Thunb.) Vahl and *Forsythia viridissima* Lindl.extract

Although both dried fruits of *Forsythia viridissima* Lindl. and *Forsythia suspensa* (Thunb.) Vahl have traditionally been considered and used as Forsythiae Fructus in Korea, the major bioactive constituents are not parallel. Thus, dried fruits of *Forsythia viridissima* and *Forsythia suspensa* may cause plant misidentification, and the scientific name (including the family) should be used. The major components of the *Forsythia viridissima* include arctiin, matairesinol, and arctigenin, and the major component of *Forsythia suspensa*l is forsythoside A (FSA) (Dong et al., 2017; Wang et al., 2018). Yi et al. reported that the aqueous extract of *Forsythia suspensa* fruits (EFSF) and its major ingredient, FSA, demonstrated an effective neuroprotective function against oxaliplatin-induced neurotoxicity *in vitro* and *in vivo* and did not interfere with anti-tumor activity (Yi et al., 2019b). Previous studies have shown that *Forsythia suspensa* and FSA exhibit significant anti-inflammatory and antioxidant pharmacological and biological activities (Cheng et al., 2014; Gong et al., 2021). Therefore, the regulatory mechanisms may be associated with oxaliplatin-induced neuroinflammation, oxidative stress, mitochondrial dysfunction, or axonal degeneration. On the other hand, *Forsythia viridissima* fruits (FVF) also recognized as a traditional Chinese medicine component, are widely known in Asia (Wang et al., 2018). Modern pharmacology studies have shown that FVFdemonstrates various pharmacological activities, including antioxidant and anticancer activities (Bao et al., 2016; Lee et al., 2017). To assess the neuroprotective effects of the aqueous extract of *Forsythia viridissima* fruits (EFVF) and its major constituents, Yi et al. (2019a) characterized and quantified the chemical constituents of EFVF using an ultra-high performance liquid chromatography (UHPLC)-diode array detector method, and sequentially identified three major constituents in EFVF, namely arctiin, matairesinol, and arctigenin. Finally, the results of the *in vitro* cytotoxicity and *in vivo* neuroprotective activity indicated that EFVF not only exerted a protective effect on OIPN, but could also support preventive effects. Among the major constituents of EFVF, arctigenin and matairesinol play a protective role against peripheral neurotoxicity. EFVF can confer protection to DRG cells against oxaliplatin-induced mitochondrial membrane depolarization, recovery of apoptosis caused by mitochondrial membrane dysfunction. Therefore, both *Forsythia viridissima* and *Forsythia suspensa* could be considered a useful herbal medicine for the treatment of OIPN in cancer patients.

#### 4.1.2 EGb761

EGb761 is a standardized extract of Ginkgo biloba L. leaves, and reportedly exhibits antioxidant properties as a free radical scavenger in the central nervous system (Diamond et al., 2000). To investigate and validate the neuroprotective properties of EGb761 on the peripheral nervous system, Oztürk et al. conducted *in vivo* experiments on peripheral neuropathy induced in mice by cisplatin administration (Oztürk et al., 2004). In their experiments, the authors found that EGb761 could confer antioxidant protection to primary sensory neurons against cisplatin-induced peripheral neuropathy. Specifically, EGb761 could alleviate symptoms of cisplatin-induced peripheral neuropathy in mice and retain primary sensory neurons morphology and capacity to regenerate, which is the direct demonstration of the effect of EGb761. Although EGb761 is a standardized extract (containing approximately 24% flavone glycosides and 6% terpene lactones), full details (such as the type and concentration of extraction solvent) regarding the handling of the extract do not be provided in the original study. Multiple antioxidant protection of EGb761 such as scavenging of peroxyl radicals, superoxide anions and nitric oxide, and inhibition of xanthine oxidase activity may have rescued cisplatin-induced oxidative damage in several types of cells including neurons.

#### 4.1.3 *Vitis vinifera* L. Hydroalcoholic Extract

*Vitis vinifera* L. (commonly grape) has been widely consumed as a food and beverage and has been used as a traditional medicine against various diseases worldwide since ancient times (Labanca et al., 2020). *Vitis vinifera* extract as medicinal plant dietary supplements has various biological activities, it is the responsibility of the scientific community to obtain strict and reliable information on the experimental and clinical pharmacology of such products. Therefore, it has been intensively investigated in terms of its chemical composition and biological activities (Shi et al., 2003; Fernandes et al., 2015). Recently, a study showed that *Vitis vinifera* hydroalcoholic extract could help prevent oxaliplatin-induced oxidative stress and neuropathy *in vitro* and *in vivo* by reducing cell mortality, oxidative damage, and apoptosis induced by oxaliplatin. It has been reported to also play a positive role in maintaininganti-tumor efficacy (Micheli et al., 2018). *In vivo*, *Vitis vinifera* extract significantly reduced the oxaliplatin-dependent superoxide anion increase and lipid peroxidation in rat astrocytes. This inhibitory effect of *Vitis vinifera* on astrocytes can be considered one of the aspects at the base of its neuroprotective and anti-hyperalgesic effect. On the other hand, oxaliplatin treatment increased Nuclear factor erythroid-2 related factor 2 (NRF2) protein level, while this effect was normalized by the *Vitis vinifera* extract.

#### 4.1.4 *Hypericum perforatum* L. Perforatum Extract

*Hypericum perforatum* L. (Saint John’s Wort) is one of the most widely prescribed agents in natural remedies and has been used for centuries worldwide (Menegazzi et al., 2020). Notably, although it is a famous medicinal plant, this plant is not absolutely safe. It has been reported that the herb-drug interaction may cause life-threatening events (Soleymani et al., 2017). Thus, *Hypericum perforatum* products should provide enough information regarding the possible risk of interaction. Nazıroğlu M and co-workers found that *Hypericum perforatum* extract could play a role in the onset and progression of neuropathic pain in rat DRG neuron via modulation of oxidative stress and Ca^2+^ mobilization through TRPM2 channels (Nazıroğlu et al., 2014). Although the precise mechanisms of oxaliplatin-induced neuropathy are complex and not fully elucidated, oxidative stress as an essential mechanism in a rat model of painful oxaliplatin-induced neuropathy has been suggested (Di Cesare Mannelli et al., 2013b). In a subsequent study, Cinci et al. suggested that *Hypericum perforatum* extract not only exerted a significant antioxidant effect in an *in vitro* model of oxaliplatin-induced neurotoxicity, but also reduced caspase-3 activity in rat astrocytes (Cinci et al., 2017). As the extract decreased both the presence of oxygen reactive species and oxidative stress-induced damage to biomolecules, and did not reduce the cytotoxicity of oxaliplatin, it could be expected to be established as a new strategy for the treatment of chemotherapy-induced neuropathy. However, considering the herb-drug interaction of *Hypericum perforatum*, we need to be vigilant when using medicinal plants, especially in specific conditions and specific populations.

#### 4.1.5 *Astragalus mongholicus* Bunge Extract

*Astragalus mongholicus* Bunge is one of the most widely used traditional Chinese herbal medicines. The total extract of *Astragalus mongholicus* contains several important bioactive ingredients, such as polysaccharides, saponins, and flavonoids (Huang et al., 2013; Xu et al., 2014; Xu et al., 2021). It demonstrates outstanding antioxidant properties and significant cytoprotective properties (Toda and Shirataki, 1999; Guo et al., 2019). The neuroprotective properties of the diverse *Astragalus mongholicus* extracts, an aqueous and two hydroalcoholic (20% and 50% HA) extracts, were evaluated in oxaliplatin-treated neurons by Di Cesare Mannelli L et al. (Di Cesare Mannelli et al., 2015; Di Cesare Mannelli et al., 2017b). These studies suggested that aqueous and 50% hydroalcoholic extracts demonstrated protective properties. Among them, 50% hydroalcoholic extracts played an essential role in preventing the activation of the apoptotic enzyme caspase-3. Both aqueous and 50% hydroalcoholic extracts exerted significant antioxidant effects, reducing morphometric and molecular alterations induced by oxaliplatin in the peripheral nerves and DRG. Simultaneously, it is a specific and crucial issues that whether the different extraction method of *Astragalus mongholicus* (aqueous, and two hydroalcoholics, 20%HA and 50%HA) affect drug concentration and bioavailability. Astragaloside IV (AS-IV) is one of the major active components of *Astragalus mongholicus* (Gong et al., 2018). It has been reported that this drug exerts a neuroprotective effect by suppressing oxidative damage-induced neuronal apoptosis in nervous system disease (Kim et al., 2015). Recently, Xu et al. examined the neuroprotective mechanism of AS-IV in a rat model of oxaliplatin-induced neurotoxicity. The experimental results verified that AS-IV could regulate neuroinflammation and could alleviate oxidative injury to improve oxaliplatin neurotoxicity (Xu et al., 2021). Therefore, *Astragalus mongholicus* could be used as a therapeutic adjuvant for oxaliplatin-induced neuropathy against etiological factors and resulting maladaptive plasticity.

#### 4.1.6 *Agrimonia eupatoria* L. Extract

*Agrimonia eupatoria* L. are mainly distributed throughout the temperate regions of the northern region and have been used in traditional medicine in Europe, Japan, Korea, and China (Granica et al., 2015). Pharmacological studies reported on *Agrimonia eupatoria* extract have demonstrated a wide range of biological properties, such as antioxidant, anti-inflammatory, and analgesic (Santos et al., 2017). To further investigate the analgesic effect of *Agrimonia eupatoria*, Lee KH et al. (Lee and Rhee, 2016) detected its activity in a rat model of cisplatin neuropathy. Compared to control animals in a cisplatin-induced neuropathic model, *Agrimonia eupatoria* extract showed significant differences and superior activity in the pin-prick test, plantar test, and paw-withdrawal threshold test. A recent study reported on *Agrimonia eupatoria* extract showed that its analgesic molecular mechanisms were associated with the downregulation of TNF-α and mitogen-activated protein kinase (MAPK) protein expression, as well as with the inhibition of the release of NO, prostaglandin E2 (PGE2), leukotriene B4 (LTB4), and interleukin 6 (IL-6) (Feng et al., 2021). Therefore, *Agrimonia eupatoria* may be deemed a potential therapeutic agent for cisplatin-induced neuropathic pain.

#### 4.1.7 *Lithospermum erythrorhizon* Siebold and Zucc. Extract

*Lithospermum erythrorhizon* Siebold and Zucc. demonstrates diverse activities and has been used in traditional medicine to treat diverse diseases, with the exhibition of anticancer, antioxidant, and neuroprotective effects (Wang et al., 2014; Lee et al., 2016). In a preliminary study, Cho et al. suggested that the aqueous extract of *Lithospermum erythrorhizon* (the dose range 25–100 μM) could be used to efficiently salvage cells from oxaliplatin-induced neurotoxicity in *in vitro* cell-based assays. Notably, one of the main advantages *in vitro* studies is that it can test a broad range of high drug concentrations that cannot be reached *in vivo*. However, if a high concentration (e.g., ≥25 μM) is used, it includes effective scientific justification. Based on this study, Cho et al. further assessed the *in vivo* efficacy of *Lithospermum erythrorhizon* (the dose range 250 mg/kg) using a neuropathic animal model. Results of the study indicated that on one hand, *Lithospermum erythrorhizon* could reduce spinal activation of microglias and astrocytes and attenuate oxaliplatin-induced mechanical hypersensitivity, while on the other hand, *Lithospermum erythrorhizon* might exert an anti-inflammatory activity in neuronal immune cells to attenuate OIPN. Therefore, these results proposed that *Lithospermum erythrorhizon* could efficiently attenuate oxaliplatin-induced neurotoxicity in both *in vitro* and *in vivo* models without affecting the antitumor properties (Cho et al., 2016).

#### 4.1.8 *Salvia officinalis* L. Extract

Members of the genus *Salvia officinalis* L., the largest genus in the Lamiaceae family, are not only used in flavoring, but are also used in traditional folk medicine worldwide (Jakovljević et al., 2019). Both *in vivo* and *in vitro* studies have confirmed that several *Salvia officinalis* species contain a substantial number of bioactive compounds (Miroddi et al., 2014; Lopresti, 2017). A few studies have suggested that the effective constituents are isolated from the components of *Salvia officinalis* and demonstrate high antioxidant and anti-inflammatory properties. Its antioxidant potential were provend both in the lipid peroxidation inhibition test and anti-radical tests. And then the anti-inflammatory properties were achieved by inhibiting the molecular targets of pro-inflammatory mediators PGE2 in inflammatory responses (Hohmann et al., 1999; Bonesi et al., 2017; Grzegorczyk-Karolak et al., 2020). *In vivo* studies demonstrated that an aqueous extract of *Salvia officinalis* exhibited potent anti-inflammatory effects in mice. Although the aqueous extract of *Salvia officinalis* exerts a somewhat weaker initial analgesic influence in comparison with morphine, it is able to effectively control the inflammatory effects of cisplatin and markedly alleviating neuropathic pain after subjection to cisplatin chemotherapy (Namvaran-Abbas-Abad and Tavakkoli, 2012).

#### 4.1.9 *Matricaria chamomilla* L. Hydroalcoholic Extract

*Matricaria chamomilla* L. (MC), a member of the Asteraceaefamily, is one of the most widely used medicinal plants (Singh et al., 2011). In traditional medicine, MC is used in sedation, pain management, and as an antioxidant agent. Notably, the analgesic and anti-inflammatory effects of MC extract have been confirmed in human research (Mao et al., 2016; Zargaran et al., 2018; Franco et al., 2020). In a mouse model of cisplatin-induced neuropathy, MC hydroalcoholic extract was found to decrease cisplatin-induced pain and inflammation and was superior to morphine in terms of anti-inflammatory effects in the second phase (Abad et al., 2011). This is mainly because MC extract can decrease production of cytokines from lipopolysaccharides *in vivo* and *in vitro* and control inflammation.

A further issue in the study of plant-derived medicines is the need for testing a positive control, and also consider the magnitude of the effect and its possible clinical relevance. Based on the above studies, the researchers found that the aqueous extract of *Salvia officinalis* and MC hydroalcoholic extract were superior to morphine (positive control) in terms of anti-inflammatory effects. Unfortunately, in many published papers, appropriate controls are ignored.

#### 4.1.10 *Aconitum carmichaelii* Debeaux Extract

*Aconitum carmichaelii* Debeaux (Buja in Korea; known as Bushi in Japanese, a processed *Aconitum carmichaelii* in China), is a frequently used herbal medicine in East Asia. *Aconitum carmichaelii* contains a range of biological ingredients and exerts various pharmacological effects, such as analgesic effects (Shibata et al., 2011). Furthermore, the analgesic effect of *Aconitum carmichaelii* on oxaliplatin-induced neuropathic pain has been reported in a clinical study (Yamada et al., 2012). Additionally, a recent study conducted using a rat model reported that oral administration (diluted with distilled water) of *Aconitum carmichaelii* demonstrated excellent anti-allodynic effects against oxaliplatin-induced cold and mechanical allodynia. On one hand, it is associated with the inhibition of activation of astrocytes; on the other hand, it is related to the downregulation of expression of proinflammatory cytokines (IL-1β and TNF-α) in the spinal cord (Jung et al., 2017). Thus, oral administration of Aconitum carmichaelii could be an alternative therapeutic option for the management of OIPN. Based on a tradtional use, most herbal remedies are administered orally, so the oral route of administration is preferred in pharmacological translational experiments related to natural products. Unfortunately, in a small part of the published original studies, this requirement is ignored.

#### 4.1.11 *Camellia sinensis* (L.) Kuntze Extract

Consumption of *Camellia sinensis* (L.) Kuntze is popular in East Asian culture, and the interest in *Camellia sinensis* consumption and its associated benefits in the Western world continues to increase (Graham, 1992). *Camellia sinensis* has attracted more attention recently in both the scientific and consumer communities. Several epidemiological studies have revealed that the consumption of *Camellia sinensis* is beneficial for patients with cancer, cardiovascular, and neurological diseases (Zaveri, 2006). The protective function of *Camellia sinensis* and its constituentsis mainly mediated by anti-oxidative and anti-apoptotic properties and via modulation of inflammatory responses (Rameshrad et al., 2017). Lee et al. conducted an animal experiment to determine whether *Camellia sinensis* extract played a vital role against oxaliplatin-induced neurotoxicity. The results of this study suggest that *Camellia sinensis* extract is a useful adjuvant to alleviate mechanical allodynia or to decrease thermal response, and may be associated with alleviation of oxaliplatin-induced acute dysfunction of axonal Na (+) channels (Lee et al., 2012). Accordingly, *Camellia sinensis* extract may be a useful therapeutic adjuvant to alleviate allodynia sensory symptoms induced by oxaliplatin administration. While the experimental results on *Camellia sinensis* extract are of potential interest, it should be emphasized that the research is still at a very early stage and any extrapolation to clinical applications must be cautious.

The investigations conducted with single medicinal plant extracts for PIPN suggested that animal models may provide basic research for further studies. However, the basic research do not guarantee that the medicinal plant extracts will be efficacious in human clinical trials. Simultaneously, single medicinal plant extracts can be composed of multiple active compounds, and for certain active compounds in extracts, their effects are unknown. Moreover, medicinal plants are not absolutely safe, such as *Hypericum perforatum* (Saint John’s Wort), it has been reported that the herb-drug interaction may cause life-threatening events. Hence, medicinal plants research should be carried out with the same meticulous care as any other medical research.

### 4.2 Plant Secondary Metabolites

#### 4.2.1 Phenols

##### 4.2.1.1 Curcumin

Curcumin, a yellow pigment-exhibiting component obtained from turmeric (*Curcuma longa* L.), is a natural phenolic compound. It is commonly used as a natural coloring agent and as a natural food additive (spice) (González-Salazar et al., 2011). Curcumin has been reported to exert antioxidant, chemo-preventive, and neuroprotective effects (Yang et al., 2020). In recent years, increasing efforts have been engaged to reveal the pharmacological mechanisms underlying the neuroprotective effects of curcumin. Previous studies have reported that this compound plays a remarkable neuro regulatory role in different types of neurotoxicity (Cole et al., 2007; Al Moundhri et al., 2013; Bachmeier and Melchart, 2019). A study conducted by Agthong et al. suggested the favorable biological effects of curcumin exerted on both functional and structural abnormalities in cisplatin-induced neuropathy (Agthong et al., 2015). These biological effects showed on that curcumin significantly ameliorated thermal hyperalgesia, sciatic motor nerve conduction velocity and the myelin thickness in the sciatic nerve. Depending on the antioxidant effects of curcumin, it is conceivable that its favorable impact on cisplatin neuropathy is likely mediated by a reduction in oxidative stress. Moreover, curcumin is provided not only in cases with cisplatin-induced neurotoxicity, but also for OIPN. In an *in vitro* study performed by Al Moundhri et al., the changes in plasma neurotensin and sciatic nerve platinum concentrations were found to be reversed, while the sciatic nerve histology in the platinum-treated rats was markedly improved viaoral curcumin consumption (Al Moundhri et al., 2013). In our study, curcumin is the only compound that both can influence cisplatin and oxaliplatin-induced peripheral neurotoxicity. Unfortunately, it is also characterized by the lack of bioavailability and problematic drug delivery, representing a series of obstacles related to the use of this compound (Tabanelli et al., 2021).

##### 4.2.1.2 Rutin and Quercetin

As a common bioflavonoid, rutin is found in vegetables, fruits, tea, red wine, and medicinal plants (Isai et al., 2009). As a dietary supplements, there are few reports of adverse effects following supplemental rutin and quercetin intake (Andres et al., 2018). Rutin reportedly exerts marked antioxidant, anti-inflammatory, and anticancer effects (Sharma et al., 2013). Quercetin is also a bioflavonoid found widely in nature, especially in plant food sources (Pandey and Rizvi, 2009). Rutin is soluble in water and converted to quercetin once it reaches the bloodstream (Morand et al., 2000). Previous work has shown that the oxaliplatin-related peripheral neurotoxic effect seems to cause, at least partially, by oxidative stress damage in dorsal horn neurons, reflected by lipid peroxidation and protein nitrosylation. While rutin and quercetin can be used to prevent lipid peroxidation and tyrosine nitrosylation to inhibit oxaliplatin-induced chronic painful peripheral neuropathy (Azevedo et al., 2013). In preclinical models, the determination of safety is as important as the proof of effectiveness, whether it is to start clinical trials or to promote natural compounds as food supplements.

##### 4.2.1.3 Formononetin

Formononetin (FN), a naturally occurring isoflavone, is reportedly abundant in various medicinal plants and herbs, such as the roots of *Astragalus mongholicus*, *Trifolium pratense* L., *Glycyrrhiza uralensis* Fisch. and Pueraria alopecuroides Craib (Ong et al., 2019). Studies have shown that FN exhibits several types of bioactivities, including antioxidant and neuroprotective effects (Sugimoto et al., 2021). In RNA interference experiments, it has been observed that FN can confer direct prevention against OIPN through activation of the NRF2 pathway. Further expression profile sequencing demonstrated that FN played a protective role via action of the NRF2 downstream-oxaliplatin metabolism enzyme, glutathione S-transferase pi 1 (GSTP1), without influencing the chemosensitivity to oxaliplatin. In fact, these results revealed that FN played a dual protective role against oxaliplatin-induced mitochondrial dysfunction and apoptosis by enhancing cell survival through the NRF2-GSTP1 axis, while providing new insights into the mechanisms involved in OIPN (Fang et al., 2020).

##### 4.2.1.4 Cyanidin

Cyanidin is also a member of the flavonoid family, which has been reported for its potent antioxidant activity (Li et al., 2015). Many fruits, especially colored berries, and vegetables are exceptionally rich in cyanidin. They are deemed the major contributors to the pigment of fruits and vegetables (Galvano et al., 2007). Li et al. reported that cyanidin as an apoptotic inhibitor effectively blocked cisplatin-induced neurotoxicity through inhibition of ROS-mediated DNA damage and apoptosis, suggesting its potential applicability in the prevention and treatment of cisplatin-induced neurotoxicity (Li et al., 2015). Specifically, cisplatin caused ROS accumulation and DNA damage, activated p53, and subsequently induced PC12 cells apoptosis. However, pre-treatment with cyanidin effectively inhibited DNA damage, eliminate the overproduced ROS, and eventually reversed cisplatin-induced PC12 cell cytotoxicity and apoptosis through inhibition ROS accumulation.

##### 4.2.1.5 Silibinin

Silibinin was first extracted from silymarin, a unique mixture of flavonolignan compounds isolated from the seeds of *Silybum marianum* (L.) Gaertn. (Liang et al., 2018). Similar to the properties noted with respect to other flavonoids, it possesses antioxidant and free-radical scavenging properties. As already stated, oxidative stress is an important target of PIPN. It has been reported that there is a relationship between the improvement of oxidative alterations and pain relief in rats treated with silibinin (Di Cesare Mannelli et al., 2012). Repetitive administration of silibinin (starting from the first day of oxaliplatin injection until the 20th) improved motor coordination and reduced oxaliplatin-induced neuropathy pain. In the study, Silibinin, as the antioxidant compound, characterizes oxidative stress parameters in a rat model of oxaliplatin-induced neuropathy. Additionally, the hepatoprotective effects (Loguercio and Festi, 2011) as well as the anticancer activity (Rugamba et al., 2021; Zhou et al., 2021)cooperatively impart silibinin a ubiquitous profile that distinguishes the compound from other antioxidant agents used clinically for the treatment of oxaliplatin-induced neuropathy.

##### 4.2.1.6 Rosmarinic Acid

Rosmarinic acid (RA) is a natural phenolic compound found in various foods, medicinal herbs, and spices (Petersen and Simmonds, 2003). RA has been reported as a mitochondrion protective agent and demonstrates various biological activities, such as antioxidant, anti-apoptotic, and neuroprotective properties (Kelsey et al., 2010; Campos et al., 2016). Aretiet al. reported that RA not only decreased oxidative stress, improved mitochondrial function to prevent oxaliplatin-induced depletion of ATP, but also suppressed the spinal glial cell activation and the expression of inflammatory markers and activated adenylate activated protein kinase (AMPK) in peripheral nerves and DRG, thereby exerting neuroprotective effects in various disease conditions. Moreover, AMPK activation also could help in the maintenance of healthy nerve cells and DRGs to alleviate OIPN by participating in the prevention of the oxaliplatin-induced mitochondrial dysfunction and gliamediated inflammation (Areti et al., 2018).

##### 4.2.1.7 6-Methoxyflavone (6-MF)

Flavonoids, a class of polyphenolic compounds, are secondary metabolites that exist in various plants and recognized as critical components for health promotion and reductions of various disease symptoms (Yi, 2018). Flavonoids exhibit several biological properties, such as antioxidant, antitumor, and neuroprotective actions (Heim et al., 2002; Cho et al., 2013). 6-Methoxyflavone (6-MF) is a naturally occurring flavone that establishes interactions with many neurotransmitter systems (Johnston, 2015). Investigators in one study directly compared the analgesic actions of 6-MF and gabapentin in BALB/c mice and male Sprague-Dawley rats. Their results revealed that 6-MF could relieve cisplatin-induced neuropathic allodynia and hyperalgesia without the occurrence of any potential side-effect of gabapentin. The peripheral analgesic action of 6-MF is most probably mediated through the inhibition of activities of cyclooxygenase enzyme (COX-1) and its isoform cyclooxygenase 2 (COX-2), which may be considered a desirable therapeutic approach against PIPN (Shahid et al., 2017).

#### 4.2.2 Alkaloids

##### 4.2.2.1 Neoline

Plant alkaloids constitute the largest group of natural products, presenting with a broad spectrum of pharmacological activities and biological activities, especially anticancer and antioxidant effects (Efferth and Oesch, 2021). Neoline, a plant alkaloid, is one of the major active ingredients of processed aconite root (the root of *Aconitum carmichaelii*, Ranunculaceae), which is used in traditional medicine in Asia to generate thermal energy and to treat pain associated with cold stimulation in the body (Tanimura et al., 2019). With the conduction of further studies, researchers reported that neoline could inhibit Nav1.7 voltage-gated sodium channel (VGSC) currents against oxaliplatin-induced mechanical hyperalgesia and cold allodynia in mice in a concentration-dependent manner (Suzuki et al., 2016; Nakatani et al., 2020). As already stated, neoline seems to be a specific active ingredient in *Aconitum carmichaelii*, although the active components cannot be easily specified (Suzuki et al., 2016). It maybe considered as a marker compound to define the quality of *Aconitum carmichaelii* products for the treatment of neuropathic pain (Tanimura et al., 2019).

##### 4.2.2.2 *Corydalis saxicola* Bunting Total Alkaloids

*Corydalis saxicola* Bunting, a well-known traditional folk medicine in South China, has been previously shown to possess a wide range of biological activities and is used for the treatment of various hepatic diseases (Cheng et al., 2008). Its active ingredients are *Corydalis saxicola* Bunting total alkaloids (CSBTA), which primarily include dehydrocavidine, palmatine, and berberine. Moreover, CSBTA have been shown to possess anticancer and analgesic properties. (Ju et al., 2020). Recently, *in vitro* studies have revealed that CSBTA can be used to alleviate cisplatin-induced peripheral neuropathy. By comparing the density of PGP 9.5 immune positive intraepidermal nerve fiber (IENF) in different groups of rats plantar, the authors found CSBTA could markedly prevent the loss of IENFs, which has been proven to be closely related to neuronal damage and neuropathic pain. Oral administration of CSBTA could substantially reduce high levels of PGE2, IL-1β, TNF-α, TRPV1, and p-p38 induced by cisplatin in rats. In addition, different concentrations of CSBTA medication could inhibit increase of TRPV1 and p-p38 caused by cisplatin in rats. Therefore, the underlying mechanism may involve two critical aspects; on one hand, it is related to the reduction of neuronal damage and IENF loss; on the other hand, CSBTA exerts its therapeutic actions by inhibiting inflammation-induced p38 phosphorylation to block TRPV1 activation (Kuai et al., 2020).

#### 4.2.3 Terpenes

##### 4.2.3.1 TanshinoneIIA

*Salvia miltiorrhiza* Bunge (Danshen, in China) is a valuable and popular herbal medicine, with immense potential for application in the management and treatment of various ailments and conditions (Xu et al., 2018). Tanshinones, one of the active diterpenoid constituents, are the most abundant components in the roots of *Salvia miltiorrhiza*, and their antioxidant and anticancer biological activities have been extensively investigated in Asian countries (Chen et al., 2013; Chen et al., 2014). There is evidence to support the assumption that certain tanshinones (particularly, tanshinoneIIA and cryptotanshinone) exert neuroprotective and analgesic activities and the repeated administrations (10 mg/kg) highlighted the effectiveness and potency of TIIA. (Di Cesare Mannelli et al., 2018). In this context, the protective effect of tanshinoneIIA on oxaliplatin-induced neurotoxicity was demonstrated in animal models reported by Cheng et al. (2019). This mechanism of action might be related to the prevention of excessive oxidative stress via reduction of ROS levels and mitochondrial membrane potential loss. In the study, they also found that autophagy was suppressed upon oxaliplatin treatment, which was partly attributed to the inhibition of the PI3K/AKT/mTOR pathway. Lastly, the addition of tanshinone IIA released the inhibitory effect of oxaliplatin on autophagy through the PI3K/Akt/mTOR signaling pathway.

##### 4.2.3.2 Ginsenoside F2

*Panax ginseng* C.A.Mey. is a famous medicinal plant that has been used as a panacea for more than 2,000 years in the medical systems of Kampo and traditional Chinese medicine (Chen et al., 2018a). In recent decades, numerous studies have focused on the use of *Panax ginseng* to realize its biological benefits on human health (Boopathi et al., 2020). Ginsenosides (triterpene saponins) are reportedly the major active compounds of *Panax ginseng* and belong to the saponin family, including ginsenosides F1, F2, Rd, Rg3, Rh1,Rh2, and more than 30 active components (Yoon et al., 2012). Ginsenoside F2 was found to exhibit significant preventive effects on oxaliplatin-induced decreases in neurite-like outgrowth in differentiated PC12cells (Suzuki et al., 2015). Previous studies have revealed that ginsenoside F2, as a natural compound, plays a potential role in ameliorating inflammation by inhibiting the generation of IL-17 and ROS in γδT cells and neutrophils, respectively (Park et al., 2016). Accordingly, it maybe speculated that the mechanism may be similarly involved in the neuroprotective effects exerted by ginsenoside F2 against OIPN.

##### 4.2.3.3 Ginsenoside Rg3

Similar protective effects of ginsenoside F2 have been observed in other active components of *Panax ginseng* . A recent study has revealed that ginsenoside Rg3 also confers protective effects against oxaliplatin-induced neurite damage and can be used effectively to provide relief in cases of oxaliplatin-induced neuropathic pain in mice (Suzuki et al., 2017). This maybe related to their involvement in the regulation of ion channels and receptors, and some studies have revealed that ginsenoside Rg3 stabilizes excitable cells by preventing the influx of cations such as Ca (2+) and Na (+) (Nah, 2014). Altogether, these results demonstrate that ginsenoside Rg3 and ginsenoside F2 may be considered promising agents for application in the prevention and treatment of oxaliplatin-induced neuropathies.

#### 4.2.4 Sulfur-Containing Compounds

##### 4.2.4.1 Ergothioneine

Ergothioneine (ET) is mainly derived via consumption of various mushrooms, which were considered to be valuable natural sources of ET. (Tsiantas et al., 2021). As a natural bioactive product, ET demonstrates strong antioxidant and neuroprotective capabilities (Yang et al., 2012; Halliwell et al., 2018). Oxaliplatin-induced peripheral neuropathy has been reported to be caused by oxaliplatin accumulation in the DRG neurons. The organic cation transporter novel1 (OCTN1) is one of the transporters responsible for accumulation in DRG neurons (Jong et al., 2011). Additionally, a previous study conducted in 2005 by Lamhonwah et al. demonstrated mitochondrial expression of OCTN1 (Lamhonwah and Tein, 2020). As a substrate/inhibitor of OCTN1, ET not only mitigated OIPN in rats by decreasing the accumulation of OCTN1 in DRG neurons, but also exerted excellent preventive effects against OPIN by decreasing oxidative stress in DRG neurons (Nishida et al., 2018). Thus, ET intake might mitigate the conditions of patients presenting with OIPN and might help improve their quality of life.

##### 4.2.4.2 Alpha-Lipoic Acid

Alpha-lipoic acid (aLA) is widely distributed in nature and is mostly obtained from food, especially from fruits and vegetables (Nguyen and Gupta, 2021). Moreover, there is evidence to support the fact that aLA is a biological antioxidant found naturally in the mitochondria and leads to the enhancement of mitochondrial function (Santos and Kowluru, 2011). The authors used an *in vitro* model of chemotherapy induced neuropathy investigating the mechanisms of neurotoxicity and neuroprotection. Reports have suggested that aLA confers protection against cisplatin-induced neurotoxicity through its antioxidant and mitochondrial regulatory functions, including prevention of mitochondrial energetic failure, neuronal apoptosis, and axonal damage, with induction of the expression of frataxin (an essential mitochondrial protein with anti-oxidant and chaperone propertie) (Melli et al., 2008). And after silencing frataxin, aLA did not show neuroprotective effects against cisplatin, implying that frataxin may play a key role in neuroprotective pathways.

##### 4.2.4.3 Glucoraphanin and Sulforaphane

Hydrogen sulfide (H_2_S) has recently been recognized as the third endogenous gaseous transmitter in addition to nitric oxide (NO) and carbon monoxide, and it has attracted considerable attention in the scientific community as a biologically important signaling molecule (Wang, 2002; Chen et al., 2007). Accumulated data reveal that H_2_S exerts positive effects on the nervous system in synaptic modulation (Wallace and Wang, 2015). Recent studies have reported the H_2_S-releasing properties of natural isothiocyanates (ITCs)that are considered as potential neuroprotective drugs (Martelli et al., 2020). Natural ITCs are derived from the hydrolysis of glucosinolates. Glucoraphanin (GRA) is a glucosinolate present in *Brassica oleracea* L. seeds, while sulforaphane (SFN) is an ITC that occurs in stored form as GRA in cruciferous vegetables, especially in broccoli sprouts. GRA is converted to SFN by the action of the plant enzyme myrosinase (Fahey et al., 2001; Vanduchova et al., 2019). It was found that GRA and SFN could be used to reduce neuropathic pain by promoting the release of H_2_S and by aidingH2S-mediated activation of Kv7 channels in animal models of neuropathic pain induced by oxaliplatin exposure (Di Cesare Mannelli et al., 2017a; Lucarini et al., 2018).

#### 4.2.5 Other Kinds of Natural Ingredients

##### 4.2.5.1 Thymoquinone

Thymoquinone (TQ) is a naturally occurring bioactive phytochemical isolated from essential oils of *Nigella sativa* L. (Ibiyeye et al., 2019). TQ reportedly possesses numerous pharmacological properties, such as antioxidative, neuroprotective, and anti-carcinogenic activities. Thus, it exerts vital functions as a chemopreventive and therapeutic agent in the treatment of various diseases and conditions (Sarkar et al., 2021). Üstünet al. conducted a study (pre-treated mice with or without varying doses of TQ prior) at the animal level. They proposed that TQ could confer protection to peripheral sensory neurons of mice against cisplatin-induced neurotoxicity by promoting neuronal cell viability and neurite outgrowth in a dose-dependent manner. Mechanistically, the adverse effects of cisplatin-induced neurotoxicity were likely mitigated *via* the potent antioxidant and free radical scavenging activities of TQ. On the other hand, TQ treatment was able to inhibit the apoptotic cascade (increasing Bcl-2 expression, repressing the activation of caspase-9 and caspase-3, reducing the cleavage of PARP-1) (Goyal et al., 2017; Üstün et al., 2018).

##### 4.2.5.2 Coumarin and Cinnamic Acid

*Cinnamomum cassia* (L.) J. Presl is classified as an important drug in East Asia and is used for treating various cold-related diseases. Using UHPLC, Chae et al.have confirmed that the primary constituents of *Cinnamomum cassia* include coumarin, cinnamic acid (CA), and cinnamaldehyde (CD) (Chae et al., 2019). In a previous study, Chae HKet al. (Kim et al., 2016). demonstrated that the water extract of *Cinnamomum cassia* and coumarin could be used to attenuate oxaliplatin-induced cold allodynia by suppressing the activation of spinal astrocytes and microglia and by inhibiting the increase in spinal levels of IL-1β and TNF. In another study (Chae et al., 2019), the results of *in vivo* extracellular recordings suggested that CA played a pivotal role in the anti-allodynic effect exerted in oxaliplatin-treated rats. The action of CA is associated with the attenuation of spinal wide dynamic range neuron firing, which is increased by oxaliplatin treatment. Mechanistically, the analgesic effects of CA were similar to those of coumarin, involving the downregulated expression of glial activation and/or cytokines (IL-1β and TNF). In the study, researchers characterized and quantified the chemical constituents of *Cinnamomum cassia* (coumarin, CA and CD) by ultra-high performance liquid chromatography, while the results suggested that CA, but not CD played a pivotal role in the anti-allodynic effect. Thus, to enhance eproducible pharmacological activity, the extract must be chemically characterized (e.g., by ultra-high performance liquid chromatography).

### 4.3 Polyherbal Preparations

#### 4.3.1 AC591

AC591 is a standardized extract of HuangqiGuizhiWuwudecoction (HGWD, Ogikeishigomotsuto, in Japanese), which consists of *Astragalus mongholicus*, *Cinnamomum cassia*, *Paeonia lactiflora* Pall., fruit of *Ziziphus jujuba* Mill. and *Zingiber acuminatum* Valeton. HGWD is commonly used for treating numbness, vibration sensation, cold sensation, and in limb ache therapy, and has attracted the interest of researchers. Recently, many studies have demonstrated that HGWD exerts neuroprotective effects (Tong and Hou, 2006; Schröder et al., 2013). Cheng et al. explored the neuroprotective strategies of AC591in animal models of oxaliplatin-induced neuropathy. They found that after AC591 pretreatment in animal models, events of oxaliplatin-induced cold hyperalgesia, mechanical allodynia, and morphological damage were decreased. This protective function largely relies on the modulation of multiple molecular targets and pathways that participate in the downregulation of expression of pro-inflammatory cytokines (such as IL-1β, IL-6, and TNF-α) (Cheng et al., 2017).

#### 4.3.2 Gyejigachulbu-Tang

Gyejigachulbu-tang (GBT) (Gui-Zhi-Jia-Shu-Fu-Tang, Chin. Keishikajutsubuto, Jap.) is a herbal formula containing *Cinnamomum cassia*, *Paeonia lactiflora*, *Atractylodes lancea* (Thunb.) DC., fruit of *Ziziphus jujuba*, *Glycyrrhiza uralensis* Fisch., *Zingiber acuminatum*, and *Aconitum carmichaelii*. In East Asian countries, such as Korea, Japan, and China, GBT has been widely used to treat various pain symptoms (Ahn et al., 2014). Many lines of evidence have demonstrated that the morphology and function of spinal astrocytes and microglia change after the occurrence of peripheral nerve injuries, which may directly influence the synaptic transmission and excitability of neighboring neurons, leading to behavioral hypersensitivity (Miyoshi et al., 2008; Inoue and Tsuda, 2018). Ahn et al. suggested that GBTcould be used to effectively relieve oxaliplatin-induced acute cold and mechanical hypersensitivity. This protective effect was achieved via prevention of the activation of spinal astrocytes and microglia, as well as restoration of immune activities of glial fibrillary acidic protein (GFAP) and OX42 (microglia marker) (Ahn et al., 2014).

#### 4.3.3 Danggui Sini Decoction

Danggui Sini decoction (DSD) is prepared using an aqueous extract of *Angelica sinensis* (Oliv.) Diels, *Cinnamomum cassia* and *Pueraria alopecuroides*. It has been used to treat neuropathic pain (Liu et al., 2017) and has been proven to be effective in inhibiting oxidative stress and apoptosis (Chen et al., 2018b). A previous study presented comprehensive evidence of the neuroprotective effect of DSD in OIPN. The study results indicated that after DSD treatment, the current amplitude of DRG cells undergoing agonist stimuli, the inflammatory response was significantly reduced and the numbers of Nissl bodies and ultra-micro structures of DRG cells were significantly improved. Collectively, it was evident that DSD could confer protection against neurotoxicity of OIPN in rats by inhibiting the development of inflammatory lesions, reducing the current amplitude of DGR cells undergoing agonists stimuli (TRPV1 agonist, TRPM8 agonist, and TRPA1 agonist). While it also could improve ultra-microstructures and increase the number of Nissl bodies to prevent against neurotoxicity of OIPN (Ding et al., 2020).

#### 4.3.4 Goshajinkigan

Goshajinkigan (GJG, in Japanese), also known as Niu Che Sen Qi Wan (in Chinese), and Jesengsingi-Hwan (in Korean), is composed of 9 crude medicinal plants and a kind of poria sclerotium in fixed proportions (*Rehmannia glutinosa* (Gaertn.) DC., *Achyranthes bidentata* Blume, *Cornus officinalis* Siebold and Zucc., *Paeonia suffruticosa var. papaveracea* (Andrews) A. Kern., *Alisma plantago-aquatica* L., *Dioscorea polystachya* Turcz., *Plantago asiatica* L., *Poria cocos* (Schw.)Wolf, *Aconitum carmichaelii* and *Cinnamomum cassia*), each of which contains several bioactive ingredients (Cascella and Muzio, 2017). It has been investigated for the treatment of multiple neurological symptoms, such as numbness and pain (Tawata et al., 1994; Kono et al., 2011). GJG has been the most studied herbal formula for the prevention of OIPN in case studies and clinical trials (Nishioka et al., 2011; Kono et al., 2013; Oki et al., 2015). The potential underlying mechanisms of action of GJG on oxaliplatin-induced cold hyperalgesia and mechanical allodynia have been suggested in a rodent model of neuropathy (Ushio et al., 2012). These findings demonstrated that GJG prevented oxaliplatin-induced acute peripheral neuropathy by suppressing the functional alteration and mRNA overexpression of TRPM8 and TRPA1 in the DRG (Kato et al., 2014; Mizuno et al., 2014). Furthermore, in another study, Mizuno et al. elucidated the benefits and underlying mechanisms of combination therapy with GJG and *Aconitum carmichaelii* (Mizuno et al., 2016). The aforementioned study indicated that GJG attenuated OIPN by counteracting the sensitization of nerve fibers in the peripheral nervous system (Aδ-and Aβ-fibers)and exerted a significant analgesic effect against cold hypersensitivity and mechanical allodynia. Notably, *Aconitum carmichaelii* potentiated these protective effects.

As these polyherbal preparations are Asian herbal combination remedies and have been trialled in Asian (such as in Japan, China, Korean), it may be difficult to adapt to other countries according to their national regulatory agencies. Another limitation with polyherbal preparations is that for certain medicinal plants, their effects are also unknown. Thus, it is difficult to clearly identify all details of its mechanism of action. Herewith, the current published papers show that there is limited evidence for the concurrent administration of polyherbal preparations as adjuvants to peripheral neurotoxic agents for the management of PIPN.

## 5 Summary and Perspectives

This review covers the most comprehensive information reported on natural products that interfere with PIPN (Table 1, Table 2). It is evident that single phytochemicals and medicinal plants occupy a major proportion of the natural products that interfere with PIPN, as presented in this study; herbal combinations account for a less considerable fraction. This may be attributed to the fact that traditional scientific studies are based on the investigation of a single agent or active compound; however, the main problem in studies involving herbal combinations is the elucidation of their mechanism of action and the fact that each individual medicinal plants extract contains several active ingredients. Moreover, although herbal combinations demonstrated a remarkable synergy in Asian traditional herbal therapies, they also complicated study analysis, interpretation of the data, and assessment of benefit (Schloss et al., 2017). For instance, as mentioned above, GJG, a herbal combination, has been extensively studied in laboratory and animal or human *in vivo* experiments and has demonstrated neuroprotection properties. However, a placebo-controlled, double-blind, randomized phase III study conducted to compare fluorouracil, leucovorin, and oxaliplatin (mFOLFOX6), with and without GJG, failed to meet its primary endpoints. In the study, the primary endpoint of clinical study was the time to grade 2 or greater sensory neuropathy, as measured according to the National Cancer Institute Common Terminology Criteria for Adverse Events (NCI CTCAE, version 3.0). In the interim analysis, the incidence of grade 2 or greater neurotoxicity was 50.6% in the GJG group and 31.2% in the placebo group. A Cox proportional hazards analysis indicated that the use of GJG was significantly associated with the incidence of neuropathy. GJG did not prevent oxaliplatin-associated peripheral neuropathy in this clinical trial. The clinical study was therefore terminated. Therefore, it is not currently recommended in standard practice (Oki et al., 2015; Kuriyama and Endo, 2018). Similarly, results derived from empirical identification of agents and interventions for mitigating PIPN have been equivocal and somewhat disappointing. Currently, there is no reliable evidence to support the use of any specificagent for the treatment or prophylaxis of PIPN. Evidence on only duloxetine has been reported for application in the treatment of patients with established and painful PIPN. Despite this, its benefits have been limited.

Although natural products have shown potential for exerting protective and therapeutic benefits against PIPN in preclinical studies, it should be acknowledged that the transition from lab to clinical utility in human subjects is a distant reality (Oveissi et al., 2019). From the summary analysis, some problems still can be seen in the current research. First, specific and crucial issues related to medicinal plants include plant misidentification, lack of standardization of the extracts, failure to report the extract type, as well as whether the extraction method and source of medicinal plants affect drug concentration and bioavailability. Secondly, the current experiments are mostly studied at cells and mice. on the one hand, it is difficult to extrapolate the dose from animal experiments to human situation. on the other hand, it is uncertain that whether there is any deviation and potential safety risks in the dose and efficacy of drugs applied to human body. Nevertheless, potentially serious adverse events, including herb–drug interactions, have been described. For example, the concomitant use of *Hypericum perforatum* (Saint John’s Wort) has been reported that herb-drug interaction may cause life-threatening events. This indicates the need to be vigilant when using medicinal plants, particularly in specific conditions, such as during pregnancy and in the paediatric and elderly population. Finally, few clinical studies have been conducted on medicinal plants against PIPN, and they are subject to many confounding factors, and the therapeutic response varies greatly among individuals or the patient population as a whole. Thus, firm conclusions of efficacy cannot be generally drawn. In addition, it should be highlighted that clinical research is at a very early stage and efficacy and/or safety data of many medicinal plants ingredients are mostly based on poor-quality research. These disadvantages may explain the lack of relevant research progress in PIPN prevention and therapy strategies during the last decades (Rubió et al., 2014; Calls et al., 2020).

Moreover, exploration of new prevention and treatment strategies for PIPN has also contributed to increasing the understanding of its pathogenesis. In future, studies should be conducted not only for revealing the mechanisms of action of single ingredients and single medicinal plant, but also for elucidating the interactions established in combinations of substances, herbal formulas, and those established with platinum-based chemotherapeutics; analyses should be conducted comprehensively by considering the above-mentioned aspects.

In response to these issues, the novel nanoformulations selectively accumulate in in tissue cells owing to the enhanced permeability and retention effect, and then exert active drug release function (Nakamura et al., 2016). At present, nanotechnology has been applied in various fields of biomedical science (Heath, 2015). Green synthesis of novel nanoparticles focusing on biosafety and biocompatibility offers hope for overcoming current limitations of poor targeting, insufficient absorption, poor bioavailability (Ho et al., 2017; Ma et al., 2019). Moreover, a highly significant level of consensus framework among the experts is necessary to establish the most appropriate models and methods, as well as to establish a maximum safe dose of phytochemicals and medicinal plants for use in the human body for application in future studies. Overall, plant-derived medicines are invaluable sources for the development of natural agents with beneficial effects in the prevention and treatment of PIPN. Hence, future potential mechanism research and prospective clinical studies are essential to identify neuroprotectants with the best likelihood of success, safety, and efficacy in patients with PIPN. This process needs to be fueled through multidisciplinary exchanges, including basic and clinical researchers, academia and the pharmaceutical industry and even patient engagement.
