# Supplementary material for: Application Potential of Plant-Derived Medicines in Prevention and Treatment of Platinum-Induced Peripheral Neurotoxicity
Source: Front Pharmacol. 2022 Jan 13;12:792331. doi: 10.3389/fphar.2021.792331 (PMC8793340; doi:10.3389/fphar.2021.792331)
Supplement: Supplementary file 1 [file table1.docx]

**Supplementary materials**

**SupplementaryTable1.The structure of the plant secondary metabolites.**

| **Compound** | **Structure** |
| --- | --- |
| Curcumin | 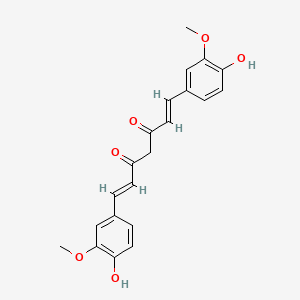 |
| Rutin | 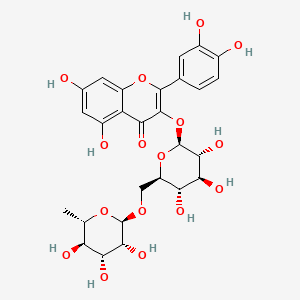 |
| Quercetin | 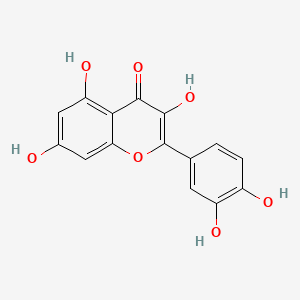 |
| Formononetin | 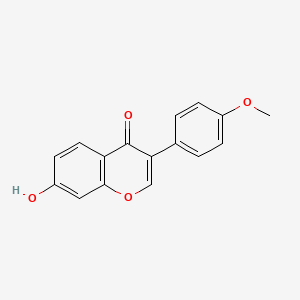 |
| Cyanidin | 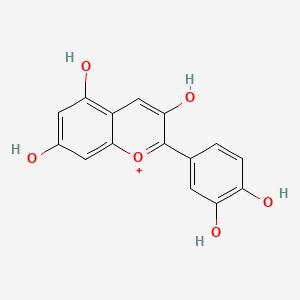 |
| Silibinin | 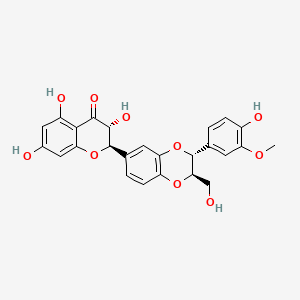 |
| Rosmarinic Acid | 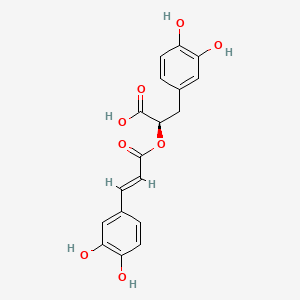 |
| Astragaloside | 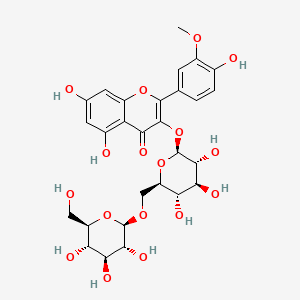 |
| TanshinoneIIA | 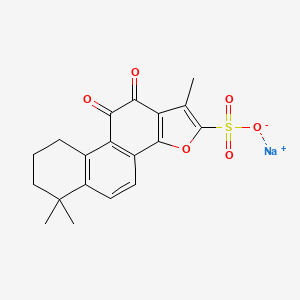 |
| Thymoquinone | 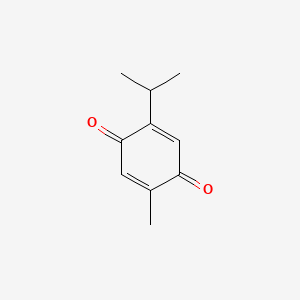 |
| Ergothioneine | 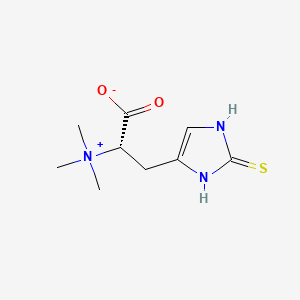 |
| Alpha-lipoic acid | 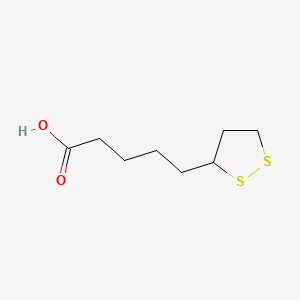 |
| 6-Methoxyflavone | 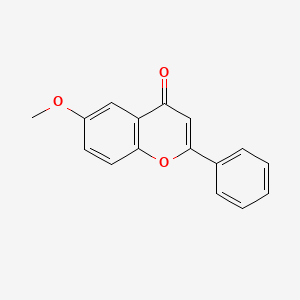 |
| Ginsenoside F2 | 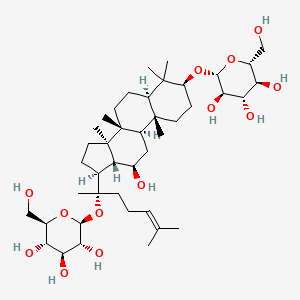 |
| Coumarin | 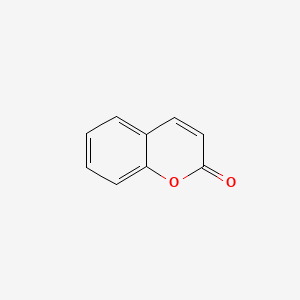 |
| Cinnamic acid | 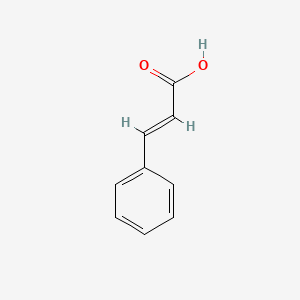 |
| Sulforaphane | 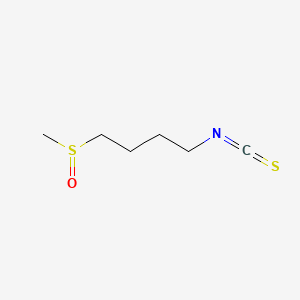 |
| Neoline | 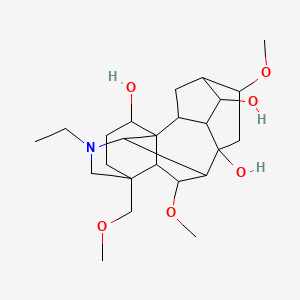 |
| Ginsenoside Rg3 | 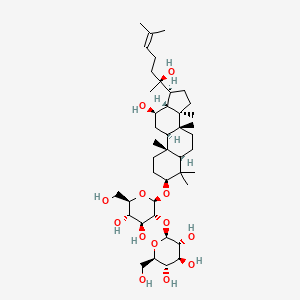 |
